# Abettors of Quackery

**Published:** 1856-02

**Authors:** 


// HALL'S JOURNAL OF HEALTH.

OUR LEGITIMATE SCOPE IS ALMOST BOUNDLESS: FOR WHATEVER BEGETS PLEASURABLE AND HARMLESS FEELINGS, PROMOTES HEALTH; AND WHATEVER INDUCES DISAGREEABLE SENSATIONS, ENGENDERS DISEASE.

VOL. III.] FEBRUARY, 1856. [NO. II.

### ABETTORS OF QUACKERY.

BE assured, ye leaders and directors in the christian world, that the most sweeping eloquence is the eloquence of example; that the most beautiful portraiture of your religion is presented in you its chiefs, standing out *alto relievo* from the common herd, towering a head and shoulders, like Absalom, above all who are about you, not in one thing, but in every, not in the *annise* and the *cummin*, but in the grand example of a whole life, reaching to remotest things in all directions. Next to the minister as a shining light, comes the editor of a religious newspaper. The minister is the representative of a congregation, the religious editor is the official representative of his sect, embracing many congregations. Death to the sleeping sentinel, remediless death, was the stern old Roman law, and what shall we say of the watchmen who stand about Jerusalem, not of the common sort who guard merely her walls, but of those more responsible, who are placed at the gates, who are found asleep, or more wicked still, are wide awake, yet publish for pay or without pay, advisedly or inadvertently, the most palpable and unblushing falsehoods. Such doings are parts of the trade of too many of the secular press, too low down in their morality for us to reach, but for you! ?

It would not be just to brand the whole religious press with publishing a falsehood, when it has only been done by a portion, and we trust, a small part. Let it be distinctly understood, we do not charge these papers with inserting these pieces knowingly; because if knowingly, they merit our contempt and have it; if unknowingly, they will feel obliged to us for bringing the repeated inadvertence to their notice.

In one of the religious papers of this city, of Nov. 17th, 1855, is the following announcement, as taken from the *New Orleans Delta.*

*Liberality of Physicians.*—" It has always been said that Physicians would disparage any remedy, however valuable, which they did not originate themselves. This has been disproven by their liberal course towards Dr. J. C. Ayres' preparations. They have adopted them into general use in their practice—this does the learned profession great credit—and we are glad to find it sustained by the liberal welcome they accord to Ayres' Pectoral and Pills. In the next column but one are two long advertisements, among the new ones, of these same remedies."

Any editor of ordinary intelligence must know, that the medical profession never did any such thing as welcome these or any other concealed remedy; and further, that no physician of any school, who had the smallest modicum of independence or self-respect, would countenance the use of any secret medicinal preparation whatever.

We have observed these kind of notices, slipped in among other items in the columns of religious newspapers for some months past. Here is another, as taken from the '*Middletown Daily Courier*' and published Nov. 8, 1855, in a religious paper at Cincinnati, Ohio.

*Marvellous cures.*—" We have always been slow to believe the wonderful cures which one medicine after another pretends to have made, but as slow as we are, we will own up, when fairly convinced. We confess our belief that Ayres' Cathartic Pills have virtues for purifying the blood, which excel any thing within the range of our acquaintance, &c. &c."

We could fill pages of this Journal with similar extracts from religious newspapers, which pass for the sayings of the editors of such papers among the mass of their readers. While we may hope that such articles get into the religious journals inadvertently, we do censure in decided terms the culpable negligence of the itemizers, resulting as it does, in misleading multitudes to tamper with health and life by the use of remedies on their own responsibility, instead of employing a regular physician, which last these same editors would be prompt to do, were they attacked with serious disease. The editors of this country are too well informed not to know, that the taking of patent medicines is the bane of the age, that it is undermining the health of multitudes. We do not here oppose newspapers advertising patent medicines for pay in the regular way of business, openly and above board, but to write editorials in commendation, to allow letters to be addressed to them, with no indication of their being, as they are, advertisements paid for at regular rates, and to admit among the reading matter for pay, commendatory extracts from other papers, when by virtue of their own intrinsic merits they would not have found a place in their columns, except by accident, is a public deception, of which an upright man would be ashamed, although he may be a mere man of the world; but for the editors of religious newspapers, who are generally clergymen, to allow such things in their columns, and then, when they get sick, to receive medical attention from the regular physician without pay, is a course of conduct, for which each reader of this journal must for himself select an appropriate epithet. But let such remember, that one of the greatest hindrances to the spread of religion is the want of a clearer line of demarcation between those who are in the church and those who are without it, and that we have no right to hope for a rapid diffusion of christianity, except in proportion as its true friends stand out from the world in beautiful distinctness in their practices, in proportion as they tower above it by the purity of their principles, and the sternness of their integrity. The brightness of their example should extend to their whole life, not in theory merely, but in practice; not in one thing, but in all.

Will it be believed that since writing the above, we have seen in a prominent religious newspaper of New York, under the general heading of "News of the Week," a paragraph following the words "BENEFACTORS OF MANKIND," going on to say: "One of our government officials, lately returned from his mission to Brazil, tells us an anecdote, that among the first inquiries made of him, was whether he knew the American Chemist, Dr. J. C. Ayer, who invented the *Cherry Pectoral* and *Cathartic Pills*, with other things of the same sort." This paper relieves itself of responsibility, by crediting the above to the *Christian Advocate*. The article further states: "It is not he who invented Brussels carpeting, or gold Brocade, whom the masses have reason to hold in regard, but he who furnishes something useful to every body," leaving the reader to infer that this is one of the items of *news of the week*, that *the Cherry Pectoral* and *Cathartic Pills* are "useful to every body;" if it had continued to say "who was willing to purchase and take them, in hurrying them out of the world," it would have been honest and true; but then, the proprietor of the paper would not have received a fee for inserting this item of "news of the week," nor would he have received pay for the long advertisement of these same articles in another part of the paper. Another peculiarity of this article credited to the "Christian Advocate" is, that it *continues* to be "news;" for *four* successive weeks. "*Benefactors of Mankind!*" Who are they—why, the man who invents a secret remedy "useful to every body"—he is Benefactor No. one—Benefactor No. two is the Itemizer or Proprietor of a religious newspaper who can be induced for a few dollars to aid and abet patent pill makers in circulating the knowledge of their remedies by the means just described.

